# Human In Vitro Skin Models for Wound Healing and Wound Healing Disorders

**DOI:** 10.3390/biomedicines11041056

**Published:** 2023-03-30

**Authors:** Elisabeth Hofmann, Julia Fink, Anna-Lisa Pignet, Anna Schwarz, Marlies Schellnegger, Sebastian P. Nischwitz, Judith C. J. Holzer-Geissler, Lars-Peter Kamolz, Petra Kotzbeck

**Affiliations:** 1 COREMED—Centre of Regenerative and Precision Medicine, JOANNEUM RESEARCH Forschungsgesellschaft mbH, 8010 Graz, Austria; 2 Division of Plastic, Aesthetic and Reconstructive Surgery, Department of Surgery, Medical University of Graz, 8036 Graz, Austria; 3 Research Unit for Tissue Regeneration, Repair and Reconstruction, Division of Plastic, Aesthetic and Reconstructive Surgery, Department of Surgery, Medical University of Graz, 8036 Graz, Austria

**Keywords:** in vitro skin models, in vitro wound healing, chronic wounds, hypertrophic scars, keloids

## Abstract

Skin wound healing is essential to health and survival. Consequently, high amounts of research effort have been put into investigating the cellular and molecular components involved in the wound healing process. The use of animal experiments has contributed greatly to the knowledge of wound healing, skin diseases, and the exploration of treatment options. However, in addition to ethical concerns, anatomical and physiological inter-species differences often influence the translatability of animal-based studies. Human in vitro skin models, which include essential cellular and structural components for wound healing analyses, would improve the translatability of results and reduce animal experiments during the preclinical evaluation of novel therapy approaches. In this review, we summarize in vitro approaches, which are used to study wound healing as well as wound healing-pathologies such as chronic wounds, keloids, and hypertrophic scars in a human setting.

## 1. Introduction

The skin represents the largest organ of the human body, and maintaining and repairing the barrier function is detrimental to the survival of any organism. Wound healing is a highly dynamic process composed of several overlapping phases that include an inflammatory response, cell proliferation and migration, extracellular matrix (ECM) deposition, and tissue remodeling [1,2,3,4]. In short, the clotting cascade is activated immediately upon injury, and hemostasis and initial restoration of the barrier integrity are assured by the formation of a fibrin clot. Concurrently, inflammatory responses are initiated, raising local and systemic host defenses against intruding pathogens and for debris clearance [1,2,5]. With subsiding inflammation, tissue regeneration is promoted by the proliferation and migration of keratinocytes, ECM deposition by proliferating fibroblasts, and angiogenesis. While the granulation tissue replaces the initial fibrin clot, keratinocytes are responsible for re-epithelialization. Finally, the wound healing process enters the remodeling phase, during which ECM components of the granulation tissue are constantly degraded and newly synthesized in order to re-establish near-normal tissue architecture and functionality [6,7,8]. The process of wound healing is tightly controlled, involving multiple cell types, each secreting numerous growth factors, cytokines, and chemokines. Perturbation of these complex physiological repair mechanisms may result in either of two major pathological outcomes, i.e. in ulcerative skin diseases [1,2,9,10] or excessive scar formation [1,2,8,11], respectively. Skin wound healing has been recognized as essential to health ever since the beginning of humankind [12], and a lot of research effort has been dedicated to investigating the cellular and molecular components involved in physiological wound healing as well as in the development of chronic wounds, keloids, and hypertrophic scars. Animal experiments were and still are frequently used to investigate the mechanisms behind physiological wound healing and pathological wound healing conditions. However, the translatability of results from animal experiments to the clinical situation has been shown to be inefficient due to a number of anatomical inter-species differences. For example, although rodent skin has more skin appendages, fewer epidermal layers, and is only loosely attached to the underlying muscle [13,14,15], rodents are often used in wound healing studies. Importantly, wound healing in rodents mainly occurs via wound contraction [16,17,18,19], which is quite different from wound healing by new tissue formation (re-epithelialization and granulation tissue formation), as observed in humans [20,21,22]. In contrast to rodents, skin morphology and physiology, as well as the wound healing processes of pigs, are more similar to the human situation [16,23,24,25]. Accordingly, several models for wound healing and wound healing pathologies have been established in the pig over time. However, it has to be mentioned here that there are also differences between these porcine models and the human situation, which also may affect the translatability of results [23,24,25]. 

**Figure 1:**
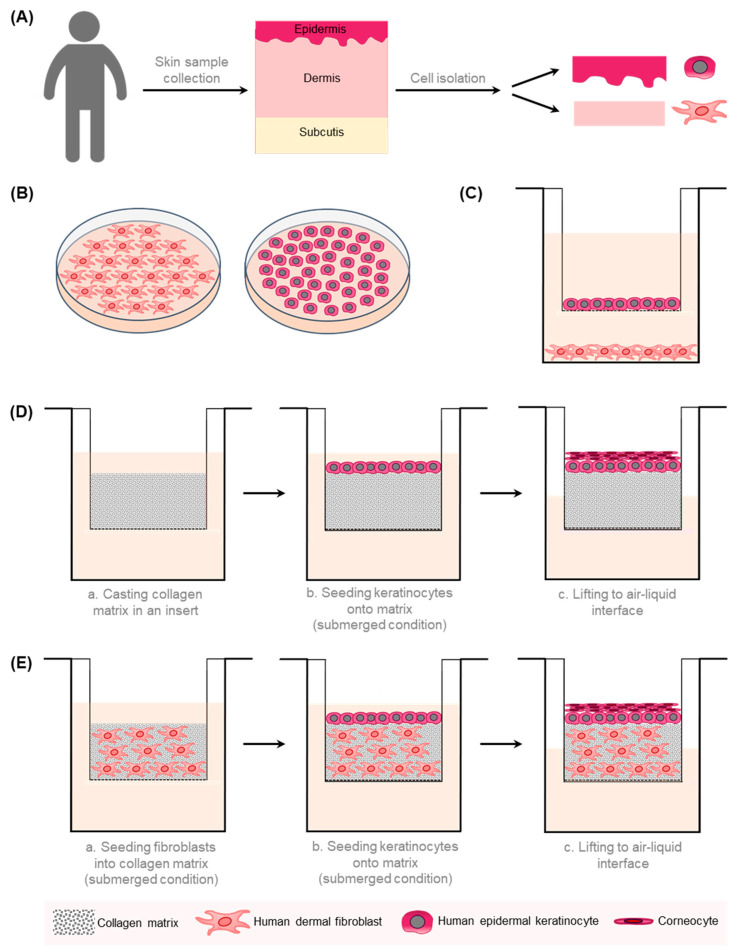
A schematic overview of the 2D and 3D in vitro models of the human skin. (**A**) Different cell types can be isolated from human skin samples. Epidermal keratinocytes are extracted from the epidermal part and fibroblasts from the dermal part of the skin. (**B**) Fibroblasts and keratinocytes are cultivated separately in a 2D monolayer. (**C**) Transwell co-culture systems comprise fibroblasts, which are grown on the well bottom, and keratinocytes, which are cultivated in a porous insert. This allows the exchange of soluble factors without direct contact between different cell types. (**D**) In the reconstructed human epidermis (RHE) model, stratified keratinocytes are cultured in a porous membrane at the air–liquid interface on top of a collagen matrix. (**E**) In a human skin equivalent (HSE), keratinocytes are cultivated atop a dermal equivalent composed of fibroblasts embedded in an ECM-like matrix [26].

Human models are highly desirable in order to improve the translatability of basic research results to the clinical situation and in order to reduce animal experiments during the preclinical evaluation of novel therapy approaches, which would be in line with the “3Rs” (Replacement, Reduction, and Refinement) principle of humane animal research [15]. The human skin is composed of three tissue layers (epidermis, dermis, and subcutaneous fat) that work together as a single organ, which is constantly changing and highly dynamic. Several approaches have been established to model human skin in vitro (Figure 1). These experimental approaches range from simple ones, such as monocultures (two-dimensional; 2D) of the dominant cell types in the skin, i.e. keratinocytes and dermal fibroblasts, over co-culture systems, to more complex three-dimensional (3D) tissue models, of the epidermis only (reconstructed human epidermis, RHE) or of dermis and epidermis (human skin equivalents, HSEs) [26]. 

More complex HSEs have been developed, which can include additional cell types, e.g., melanocytes, endothelial cells, and immune cells, and structures such as vasculature or a third layer of adipose tissue (reviewed by Hofmann et al., 2023, and in [26,27,28,29]). 

In this review paper, we present approaches available to study wound healing, chronic wounds, and excessive scarring in human in vitro models. Moreover, we will give an overview of in vitro models of pathological wound healing that have already been used to evaluate treatment options and how the results correlate to respective clinical trials. 

## 2. In Vitro Models for Wound Healing

Re-epithelialization is regarded as the hallmark of wound closure and is, therefore, the read-out for any wound healing assay. The scratch assay is a technically non-demanding and cheap, thus popular, assay, which allows studying the migration of cells on 2D surfaces. Adherent cells of interest, i.e. keratinocytes or dermal fibroblasts, are grown to a confluent monolayer, which is then deliberately “wounded” by scraping off cells, usually by means of a plastic pipette tip [30,31]. Cells migrate from intact zones of the cell layer towards the created gap until cell–cell contacts are re-established, that is, until the wound is closed. This migration is observed by bright-field imaging. Pictures are either taken repeatedly, e.g., every two to three hours, or time-lapse microscopy is used to constantly monitor cell movement. Cell migration is finally calculated by measuring the decrease of the denuded region at different times until the ‘‘wound’’ is closed [28,30,32]. Several approaches are available for wound assay analysis, starting from manually determining the distance between the wound edges over the use of freely or commercially available software identifying and calculating the “open area” to fully automated analyses of time-lapse micrographs [33]. The standard version of the scratch assay has been modified in numerous ways. Instead of the pipette tip, other strategies, such as cell scrapers or toothpicks, have been applied to induce the scratch [28]. Moreover, devices have been developed that produce highly reproducible scratches within seconds in order to facilitate high-throughput screenings [34,35]. Alternative methods to scratching have also been described, e.g. stamping, thermal, and optical (laser) wounding [28]. Plates can be coated with various ECM-components, e.g. collagen I, collagen IV, laminin, or fibronectin, prior to seeding the cells, which offers the additional possibility to analyze the migration behavior on different substrates [32]. 

The advantages of the scratch assay are obvious. It is technically easy, and standard cell culture laboratory equipment is sufficient; therefore, it is cheap. Additional instruments are only needed for time-lapse microscopy and high-throughput approaches [30,32]. One major drawback of this method is that the scratches, if performed manually, are often unevenly thick, which is likely to influence the analysis. Cells may stick to the border of the scratch, re-attach, and start migrating into the wound, leading to biased results. Additionally, scratching may mechanically destruct the plastic surface and/or the coating ECM component, which in turn may impact cell migration behavior [28,32]. 

The above-mentioned problems of the traditional scratch assay can be circumvented by using electric cell-substrate impedance sensing (ECIS^®^), a method determining parameters such as cell viability, attachment, and motility based on electric impedance measurements [36]. Cells are grown in multi-well dishes with electrodes covering the well bottoms. A constant alternating current is applied, and as cells exhibit insulating properties, an increase in impedance can be measured in real-time as cells grow and form a monolayer [37]. Wounding is performed by a pulse of high current, leading to electroporation and cell death, creating a very defined wound [38]. In this area, the impedance will drop instantly, followed by a constant increase in impedance over time, which reflects wound closure by migrating cells. The advantages of this method are the high reproducibility of wounds and automated real-time measurements. Therefore, this approach can be used in medium- to high-throughput screening experiments [39,40,41,42]. However, in contrast to the conventional scratch assay, specific equipment, i.e. the ECIS^®^ device and special electrode plates, has to be purchased. 

Scratch assays are still commonly used in drug development for the initial testing of potential therapeutic substances because they are easy to standardize and automate [28,33]. However, it has been recognized in recent decades that 2D cell culture experiments do not truly reflect physiological conditions, as input from cell–cell and cell–matrix interactions is missing, which may result in non-predictive data for in vivo responses [43,44]. The cells significantly differ from their in vivo counterparts in terms of appearance and central characteristics, such as response to extracellular stimuli, cell-to-cell interactions, morphology, gene expression, cell migration, proliferation, accessibility to nutrients and growth factors, cell signaling, and ECM synthesis [45]. The primary reasons are the non-physiological growth conditions that the cells must adapt to, such as monolayer formation and complete immersion in media. Consequently, tissue-specific arrangements that allow the interaction between cells to regulate the proliferation, differentiation, and function of cells are lost [43,44]. Moreover, the downregulation of drug-metabolizing genes is common in 2D cultured cells, and as a result, in vitro assays often fail to predict organ-specific toxicity [46]. Therefore, more complex 3D cell culture models have been developed, i.e., RHEs and basic HSEs [26]. Several protocols have been established to induce wounds in HSEs, including burn injuries, frostbite, and excisional and incisional wounds [47,48,49,50,51,52]. The culture models in 3D are not only more demanding than in 2D, but the analysis of re-epithelialization is also more laborious, as this requires the wound/skin equivalent, same as with real skin specimens, to be subjected to time-consuming histological procedures [43]. In contrast to simple scratch assays, analysis of wound healing in HSEs is not restricted to the analysis of gap closure. Additionally, morphological issues, such as the re-establishment of the basement membrane or the composition and structure of newly synthesized ECM, can be addressed [53]. Dependent on how complex the model is, the interplay of different cell types and cells and matrix/scaffold components during wound healing may be examined [54]. In order to investigate the underlying aberrant mechanisms resulting in pathological wound healing conditions and in order to test potential treatment options, the in vitro models of the skin have to be further modified. These modifications may include the use of pathological-tissue-derived cells and/or changes in media composition.

## 3. In Vitro Models for Chronic Wounds

Chronic wounds exhibit an interrupted repair process and will not heal properly within an appropriate amount of time, which is suggested to be 4 weeks to 3 months (dependent on the literature) [55]. Chronic wounds fail to progress properly through the phases of healing but are retained in a self-perpetuating inflammatory stage without transcending to the subsequent proliferative stage [55,56]. Several causative etiologies have been described, which are venous leg ulcers, arterial insufficiency ulcers, pressure ulcers, and diabetic foot ulcers [55]. Some key factors, which are critically involved in keeping a wound from healing properly, are common among these different etiologies, including an increased and/or prolonged inflammation, hypoperfusion/ischemia, and hypoxia, as well as infection and biofilm formation [55,56]. 

Most animal models of chronic wounds, e.g. the ischemic rabbit ear model, the diabetic mouse, or the skin flap ischemic wound model, to name a few, have been established by exposing an acute wound to the primary clinical causes of chronic wounds, e.g. ischemia, diabetes, pressure, or reperfusion damage [57]. Moreover, a porcine model of delayed wound healing, which is based on the induction of prolonged inflammation, has been introduced recently [58]. However, animal models have numerous limitations, including fundamental morphological and physiological differences from humans, ethical concerns, as well as economic aspects. Therefore, human in vitro models of human skin offer a valuable alternative to animal experimentation. Ideally, in vitro models of chronic wounds, which still need to be developed, would feature key aspects of chronic human wounds of different etiologies (e.g. venous leg ulcer, diabetic foot ulcer, pressure ulcer, and arterial insufficiency). Therefore, the respective skin models will be quite complex, consisting of three layers, composed of pathological-tissue-derived cells (or cell lines), comprising immune cells as well as a vasculature (at least vascular-like structures); ideally, microbiota (healthy versus pathogenic) may also be included in the model. So far, several 2D and 3D in vitro models mirroring some of the key aspects of chronic wounds have been published (Table 1).

The first steps in the development of such an ideal chronic wound in vitro model have been taken with the isolation, cultivation, and characterization of chronic-wound-derived fibroblasts because it was shown that fibroblast dysfunction is critically involved in the non-healing of chronic leg ulcers [60]. 

Various studies demonstrated that chronic-wound-derived fibroblasts exhibit an altered morphology, such as the presence of actin stress fibers and enlarged shape [59], decreased cell proliferation, as well as impaired migration ability [60,61], compared to normal dermal fibroblasts. Since primary cells are constricted in their lifespan and their use is therefore limited, an immortalized cell line retaining its pheno- and genotype during increased replication cycles is of high interest. Caley et al. developed a protocol to produce hTERT- (human telomerase reverse transcriptase) immortalized chronic wound fibroblast cell lines to provide a tool for investigating the biology of chronic wounding [62]. In addition to using chronic-wound-derived cells, modifications of the media composition are an important approach to mimicking chronic wound environment in vitro. Keratinocytes derived from a diabetic wound or non-diabetic cells cultured under hyperglycemic conditions are utilized to imitate the in vivo diabetic situation. However, inconsistent results have been reported on the use of hyperglycemic conditions, as some studies reported an inhibitory impact on the migration ability of cells [84,85,86], while other authors described just the opposite effect [87]. A study by Ueck et al. revealed that the microenvironment, such as the exact culture medium composition, as well as the age of the donor, are crucial factors for the outcome of the in vitro experiment [88].

However, limitations of such 2D in vitro monolayer cultures are the lack of cell–cell interaction or a cell-to-environment interface [63], as well as the lack of intact vasculature [88]. Therefore, 3D in vitro models are a more promising system for mimicking the in vivo situation more accurately. Fibroblasts originating from patients with diabetic foot ulcers were used to generate a 3D in vitro chronic wound model [63]. The interaction between fibroblasts and keratinocytes was assessed by creating a dermal compartment with fibroblasts seeded in a collagen type I matrix with keratinocytes seeded atop. Angiogenesis induction was identified in a tube formation assay using embedded endothelial cell-coated beads in a fibrin gel with fibroblasts seeded on top. The ability of fibroblasts to support wound closure was measured in an excisional wound healing model. Key features of chronic wounds (e.g. keratinocyte hyperproliferation, decreased revascularization, and delayed re-epithelialization) were successfully reflected in their model. Such models are an important basis for studying physiological and pathological mechanisms of the skin as well as for developing prospective therapeutics [63]. In a study by Ozdogan et al., a pre-vascularized 3D type 2 diabetic human skin model was generated. Therefore, primary cells, such as dermal fibroblasts, keratinocytes, and human umbilical vein endothelial cells, were isolated from patients with type 2 diabetes and were embedded in a hydrogel. The generated models were also successfully used as a testing platform for therapeutic materials as well as a model for evaluating the diabetic wound healing potential [64]. However, more appropriate preclinical models of non-healing wounds, especially those representing the different causative etiologies, are necessary to provide reliable predictions on the clinical success of novel therapeutic approaches.

## 4. In Vitro Models for Excessive Scarring

In human skin, two types of pathological scarring, characterized by excessive ECM deposition and prolonged granulation tissue proliferation, can be distinguished: hypertrophic scars and tumorous keloids [89,90]. Several in vitro models imitating abnormal scar formation have been established in order to investigate the basic cellular processes underlying excessive scarring and in order to provide in vitro models for testing the effectiveness of existing and novel therapeutic anti-scarring approaches. 

### 4.1. Keloids

Keloids are enlarged, raised scars that can be pink, red, skin-colored, or darker than the surrounding skin. They can develop from any wound, even after minor skin damage; they may spread beyond the original area of injury and will not regress spontaneously. The highest incidence of keloids is observed in ethnicities with darker skin. This indicates that genetic and environmental factors might be predisposing. However, the exact etiology remains elusive [91]. Keloid tissue is composed of disorganized, thick, eosinophilic collagen type I and III bundles that are randomly oriented to the epithelial surface with no nodules or excess myofibroblasts [1]. 

The development of keloids is a uniquely human trait for which no single causative gene has been identified so far. Approaches to induce the formation of keloids or keloid-like structures in animal models have been unsuccessful so far, as hypertrophic scar-like structures developed rather than keloids (as reviewed in [92,93]). The implantation of human keloid cells or tissue fragments into animal models turned out to be more successful [92,93,94,95,96,97]. However, in these models, the mechanisms leading to keloid development could be studied. The need for immunodeficient mice and the intrinsic differences between rodents and humans are further major limitations of animal models available for studying keloids [92,93]. Therefore, in vitro models are highly promising approaches, and several 2D and 3D models of keloids have been published over the years (Table 1). 

The cultivation of fibroblasts derived from keloids was first described in the late 1970s [98,99]. Similar to the in vivo situation, increased levels of collagen [98,99] and fibronectin [100,101,102] deposition, but decreased amounts of hyaluronic acid [103,104], was observed in keloid fibroblasts under in vitro conditions. The composition of the collagen, i.e. the collagen type I to type III ratio, did not differ between normal dermal fibroblasts and fibroblasts derived from keloids [105], which also corresponds to the in vivo situation. No differences were observed in the proliferation characteristics between keloid-derived fibroblasts and normal dermal fibroblasts under standard culture conditions [98,106,107]. This was unexpected given the aggressive hyperplastic phenotype observed in vivo [108,109]. Calderon et al. analyzed normal dermal and keloid fibroblasts in scratch assays and showed increased proliferation in both fibroblast populations for up to 48 h after wounding as compared to non-wounded cells [110]. Interestingly, only under these post-wounding conditions, the proliferative response was significantly greater in keloid than in normal dermal fibroblasts. Later (72–96 h), proliferation was back to normal, and no significant differences were observed between wounded and non-wounded keloid and normal dermal fibroblasts [110]. 

For over 20 years, studies have focused on the role of fibroblasts in keloid development. The role of keratinocytes in the development of keloids was first investigated in 2001 [65] based on the notion that autocrine, paracrine, and endocrine epithelial–mesenchymal interactions are essential for normal skin homeostasis, growth, and differentiation [111,112,113,114]. In their studies, Lim et al. reported that indirectly co-culturing normal and keloid fibroblasts with keloid keratinocytes increased the proliferation of the fibroblasts of either origin [65], which was confirmed by another study two years later [66]. In addition to the impact on fibroblast proliferation, keratinocyte-produced soluble factors also lead to an increased collagen deposition by the fibroblasts [115,116]. Increased motility but normal proliferation was observed for keloid-derived keratinocytes in culture alone [117]. 

An initial 3D model of keloids consisted of a dermal matrix populated with keloid fibroblasts covered by differentiated layers of normal keratinocytes mimicking the epidermal compartment [67]. In this model, increased epidermal thickness, dermal thickness, and collagen deposition; increased organization of alpha-smooth-muscle actin (a-SMA) fibers; and consequently increased contraction were observed in HSEs produced from keloid fibroblasts compared to normal fibroblast-based constructs. To further refine keloid in vitro models, a dermal model was developed containing fibroblasts of three different origins (keloid center, keloid periphery, and non-lesional skin) in a collagen continuum [68], which should allow the analysis of cell-matrix and cell–cell interactions, including the factor of fibroblast heterogeneity. In this setting, no epidermal compartment was included. RHS models containing donor-matched keloid fibroblasts and keloid keratinocytes recapitulate a number of features typical for keloids in vivo, including an increase in dermal thickness, a-SMA, and consequent contraction [69,70,71]. In order to further develop these keloid models, immunocompetent keloid models were created by including CD14+ monocytic cells from peripheral blood [72]. 

A number of studies have been performed testing current and/or promising approaches against keloid formation and progression, and we compared results from clinical in vivo studies to results from in vitro studies. The injection of glucocorticoids has been a standard first-line treatment against keloids for a long time [118,119]. Additionally, anti-proliferative agents have been investigated for the treatment of keloids. Here, 5-fluorouracil (5-FU), a pyrimidine analogue and widely used chemotherapeutic, was reported to show promising effects as a monotherapy [120] and in combination with glucocorticoids [121,122]. The main benefit of 5-FU is a significantly lower recurrence rate compared to traditional glucocorticoid treatments [123]. Studies analyzing the effects of various glucocorticoids alone or in combination on the proliferation, migration, and invasion behavior of keloid fibroblasts in vitro revealed that different glucocorticoids clearly act differentially, thus suggesting that combination therapies in vivo might be more effective [124]. Similar studies show synergistic effects of glucocorticoid treatment in combination with 5-FU, arguing for reduced dosing in keloid therapies [125]. When injected intralesionally, 5-FU leads to amelioration and flattening of keloid appearance [120,126]. An intralesional injection is associated with rare side effects that can further be minimized by adding very small amounts of glucocorticoid triamcinolone (TAC). Clinical trials showed that low-dose TAC successfully reduces local adverse effects, such as redness and ulceration, when injected in combination with 5-FU. Low-dose TAC is not expected to show any therapeutic efficacy other than diminishing the mentioned local adverse events [121,123,127]. An in vitro study by Huang et al. provided the underlying molecular basis for the clinical benefits of that combination. 5-FU induces matrix metalloproteinase 2 (MMP-2), G2 cell-cycle arrest, and apoptosis, which may be associated with p53 activation and p21 up-regulation. TAC alone only induces G1-phase arrest and poor apoptotic effects. The TAC/5-FU combination resulted in a more significant inhibition of Col-1 production in keloid fibroblast culture at 72 h after treatment compared to TAC and 5-FU alone [125]. 

Another promising treatment regime consists of surgical excision followed by adjunctive X-ray radiation. Radiation and steroid treatment also show synergistic anti-proliferative effects on steroid-sensitive keloid fibroblasts. However, a consensus on optimal radiation dosage and timing is missing. Son et al. tested different doses in cultured keloid-derived primary fibroblasts and in patients. In vitro, a maximal inhibitory effect on fibroblast proliferation was achieved at 3 Gy (~20% survival). At 9 Gy, the outgrowth from explants was completely blocked by inducing multiple cell death pathways and reducing collagen levels. Additionally, 50 kV radiation was shown to be more effective in preventing cell outgrowth than 75 kV radiation at the same dose. In patients, a single 8 Gy dose of superficial 50 kV radiation administered 34 days after keloid excision seemed to be sufficient to reduce recurrence rates. Higher radiation energy doses did not pose additional benefits [128]. The use of higher doses (15–20 Gy, rarely ≥30 Gy) is quite common in the clinical setting and greatly increases the potential risk of long-term adverse effects of radiation. Long-term follow-up (1 year) in the study by Son et al. only concentrated on the recurrence rate, and adverse effects were not questioned. 

Currently, there is no standard treatment against keloids, as most therapies are successful for only a subset of patients. Moreover, these therapies have only limited long-term effects with high recurrence rates [93]. 

### 4.2. Hypertrophic Scars

Hypertrophic scars frequently occur as a complication following cutaneous injuries, such as extensive trauma or severe burns. These scars develop approximately three months after deep injury and are characterized by a red/pink color as well as an elevated and uneven surface. Additionally, hypertrophic scars are painful, pruritic, and rigid; do not extend beyond the margins of the primary wound; and tend to subside with time, which differentiates them from keloids [1]. Myofibroblasts, which are essential for ECM formation and wound contraction, were shown to persist in hypertrophic scars, contributing to the pathologic phenotype [78,79]. 

Several animal models aiming at reproducing the clinical features of hypertrophic scars have been described in the literature (as reviewed in [129,130,131]). Apart from the ethical considerations, results from rodent models are difficult to translate to the clinical situation. Porcine models are known to be more similar to the human situation, and the red Duroc pig was especially identified to be able to develop scars very similar to human hypertrophic scars [132,133,134]. This model was recently refined by Nischwitz et al. by applying an immunomodulatory substance to full-thickness wounds, reproducibly resulting in hypertrophic scar-like tissue [135]. Although these approaches are promising, the justified ethical concerns, as well as the high costs of adequate large animal housing for long time periods, are major limitations. Preclinical studies and basic research would strongly benefit from appropriate in vitro models. 

In order to model hypertrophic scars in vitro, different approaches have been published (Table 1). Fibroblasts derived from deep dermal layers of healthy skin were reported to display characteristics similar to what was observed for hypertrophic scars, such as increased production of connective tissue growth factor (CTGF), collagen, and a-SMA, or decreased decorin amounts [74,136]. Moreover, hypertrophic scar characteristic traits, i.e., enhanced contraction, increased collagen, and a-SMA production, were observed in 3D models of dermal equivalents or HSEs using deep dermal fibroblasts [137], mechanical stress-activated fibroblasts [138], or cutaneous adipose tissue mesenchymal stem cells [139]. 

Other in vitro models relied on the use of fibroblasts originating from hypertrophic scar tissue in standard 2D cultures to analyze wound healing, proliferation, apoptosis, and migration behavior [73,74,75,76,77]. In 3D models, the implementation of fibroblasts originating from hypertrophic scars closely mimicked the in vivo phenotype [73,80,81]. While the indirect influence of the epidermal compartment was demonstrated by co-cultivating fibroblasts and keratinocytes in transwell systems [78], direct effects were observed in HSE models based on pathological keratinocytes and/or fibroblasts [82,83,140].

To date, there is no satisfactory prevention or treatment option for hypertrophic scars, mainly due to the insufficient understanding of the specific mechanisms leading to hypertrophic scars. Therefore, regarding the prevention and therapeutic approach, understanding scar formation is of utmost importance [90]. It is not possible for physicians to control systemic and genetic factors affecting the development of hypertrophic scars. The common treatment for hypertrophic scars reduces inflammation and includes silicone sheeting or gel, corticosteroid injections, radiotherapy, compression, pulsed-dye laser, and surgical procedures that reduce skin tension [141]. Khalid et al. showed in a clinical trial that the combination therapy of 5-FU and TAC is not only effective for keloids but also for hypertrophic scars [123]. 

Existent in vitro approaches to model keloids or hypertrophic scars are not yet suitable for providing reliable results for clinical application. For the development of improved therapies, deeper knowledge of the molecular mechanisms behind the progression of healing wounds to keloids and hypertrophic scars is essential. Appropriate preclinical models, ideally human in vitro models, are highly needed to elucidate the mechanisms involved in keloid and hypertrophic scar development as well as to provide a platform for the evaluation of innovative therapies. 

## 5. Limitations and Future Perspectives of In Vitro Models for Wound Healing Disorders

**Figure 2:**
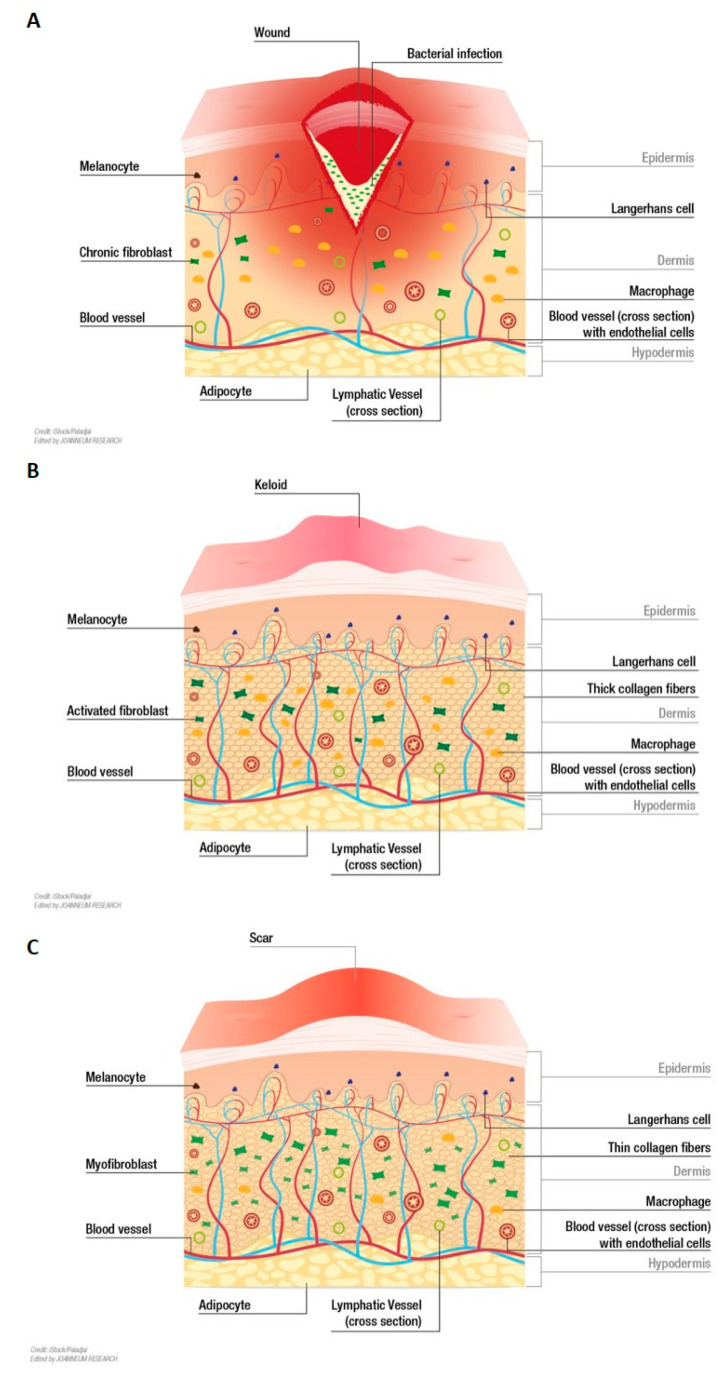
Ideal in vitro models of wound healing disorders envisioned to reproduce clinical key aspects. (**A**) Chronic wounds are characterized by an impaired repair process. A prolonged inflammatory phase, hypoperfusion/ischemia, infections, and/or biofilm formation keep chronic wounds from transcending to the proliferation phase [55,56]. (**B**) Keloids are enlarged, raised, tumor-like scars that can even extend beyond the original margins of a wound. Keloid tissue is composed of disorganized, thick, eosinophilic collagen type I and III bundles that are randomly oriented [1]. (**C**) Hypertrophic scars have a red/pink color as well as an elevated and uneven surface. In contrast to keloids, they do not extend beyond the margins of the primary wound. Hypertrophic scars are characterized by the persistence of myofibroblasts, which contribute to enhanced contraction, and increased production of collagen and a-SMA [1,78,79].

Basic in vitro models of the human skin, i.e. RHE and HSE, can be produced in quite a standardized manner nowadays (as reviewed in Hofmann et al., 2023, and in [26,27,28,29]). However, general improvements in the bi-layered “full thickness equivalents” are required to achieve a more complete in vitro model of the skin. Modeling wound healing disorders in vitro is even more demanding, as ideal models need to reliably mimic the pathological phenotype (Figure 2). The questions of isolation, amplification (by immortalization or iPSC technologies), and incorporation of patient-derived cells will be critical issues. Apart from fibroblasts and keratinocytes, ways to isolate and incorporate immune cells, endothelial cells, and adipocyte precursors in a fully patient-specific skin model will be a major challenge in the future. 

Further improvement would be achieved by the incorporation of a hypodermal compartment as a third layer would correlate better to the physiological skin anatomy, and important functions such as hormone secretion would be introduced [142,143,144]. Different approaches to include a hypodermal compartment in an HSE have been published, including the incorporation of adipose-derived stem cells [144,145,146,147], mature adipocytes [148], and native adipose tissue [149,150].

Approaches for engineering skin vasculature allowing true perfusion are a major technical issue in the field. Using a decellularized xenogenic matrix with conserved and perfusable vasculature structures [151] is one possible approach. Advances in 3D bioprinting also show promising results [152]. The fast-evolving technology of 3D bioprinting allows the manufacturing of complex biological structures using living cells, biomaterials (also called bioinks), and biological molecules as input material for layer-by-layer printing. Therefore, custom-designed tissue constructs can be produced in a highly flexible and reproducible manner [153,154]. For example, bioprinted vascular-like structures were populated with human endothelial cells or endothelial cells derived from induced pluripotent stem cells (iPSCs). This gave rise to a perfused skin equivalent that could be used for a drug delivery study [155]. 

The absence of immune cells is another major limitation of the physiological relevance of available HSEs, especially so for wound healing studies, since inflammation is driven by skin residents as well as recruited immune cells [156]. The incorporation of T-cells, dendritic cells, and/or macrophages of varying origins in skin in vitro models has been described before [157,158,159,160,161]. Moreover, immunocompetent keloid models were produced by incorporating CD14+ monocytic cells from peripheral blood [72]. However, there is still no standard source of relevant immune cells in general, especially for the generation of an in vitro model capturing all the aspects of non-healing or excessive scarring. 

The technology of iPSC allows high amounts of dedifferentiated, pluripotent cells with unlimited growth potential to be obtained from a limited number of somatic cells [162] or from non-invasive sources such as blood [163]. Thus, iPSCs are a promising source for all kinds of different cell types needed to model the full complexity of human skin. It could be shown that skin models can be generated solely from iPSCs differentiated into keratinocytes and fibroblasts [164]. Additionally, endothelial cells needed for a vascularization approach have already been derived from iPSCs [155]. With respect to patient-derived cells that are supposed to keep their pathological phenotype in vitro, the use of iPSC technology will have to be evaluated carefully. It was shown that iPSC-derived fibroblasts differed significantly from the parental fibroblasts originally isolated from diabetic foot ulcers [165]. In general, technologies such as microfluidics and bioprinting [166], in combination with innovative scaffold materials and iPSC technologies, are promising tools for the development of complex skin equivalents.

The long-term culture of HSEs is desirable for the analysis of excessive scar formation, as it is a long-term process in itself. Moreover, certain treatments with potential long-term adverse effects, such as radiation therapy, could be evaluated in preclinical studies. An in vitro skin aging [167] model has been introduced that mimics the effect of chronological aging, which is a promising starting point for the development of HSEs allowing long-term investigations. 

## 6. Conclusions

Major advances have been made to produce complex human skin equivalents in vitro, almost fully mimicking human skin morphology and functionality. Of special interest and urgently needed are in vitro models featuring human wound healing pathologies such as chronic wounds, hypertrophic scars, or keloids. Initial models based on the use of cells originating from pathological tissue have already been described. More research is needed here on how to reliably mimic the pathological phenotype and how to isolate and incorporate different cell populations originating from pathological tissue. The recent technological advances in 3D bioprinting and iPSC generation will also speed up advances in this field of research. More sophisticated and more standardized models of these wound healing pathologies would allow for the dissection of mechanistic pathways that give rise to the pathological condition. Knowledge of these pathways will speed up the development of novel (targeted) treatment options. Moreover, such models and their implementation in high-content screening procedures would also facilitate the testing and evaluation of existing and novel therapies.

## Figures and Tables

**Table 1:** Summary of in vitro models for wound healing disorders.

In Vitro Model		Cells	Medium/Matrix	Limitations	Ref.
**Chronic wounds**	2D	Chronic-wound-derived fibroblasts (venous leg ulcer; undefined)	-	Lack of cell–cell interaction/vasculature/cell-to-environment interface; not immunocompetent	[59,60,61]
		hTERT chronic wound fibroblast cell line (venous leg ulcer)	-	Lack of cell–cell interaction/vasculature/cell-to-environment interface; not immunocompetent	[62]
	3D	Fibroblasts from diabetic foot ulcers; NKs; endothelial cells	Collagen type I	Lack of vasculature; not immunocompetent	[63]
		Patient-derived (type 2 diabetes) dermal NFs; NKs; HUVECs	Hydrogel	Not immunocompetent	[64]
**Keloids**	2D	Co-culturing NFs and KFs; NKs	-	Lack of vasculature/cell-to-environment interface; not immunocompetent	[65,66]
	3D	NFs and KFs; NFs	Collagen	Lack of vasculature; not immunocompetent	[67]
		KFs of different origins	Collagen	Lack of vasculature; not immunocompetent	[68]
		Donor-matched KFs and KKs	Collagen-elastin	Lack of vasculature; not immunocompetent	[69,70,71]
		KKs and KFs; CD14+ monocytes	Collagen-elastin	Lack of vasculature	[72]
**Hypertrophic scars**	2D	HSFs	-	Lack of vasculature/cell-to-environment interface; not immunocompetent	[73,74,75,76,77]
		Hmyo and NKs	-	Lack of vasculature/cell-to-environment interface; not immunocompetent	[78,79]
	3D	HSFs	Collagen	Lack of vasculature; not immunocompetent	[73,80]
		HSFs	Fibrin	Lack of vasculature; not immunocompetent	[81]
		Hmyo, NFs, Wmyo; NKs	Manipulable sheets	Lack of vasculature; not immunocompetent	[82]
		Wmyo, Hmyo, NFs; NKs or HSKs	Manipulable sheets	Lack of vasculature; not immunocompetent	[83]

Abbreviations: hTERT: human telomerase reverse transcriptase; HUVECs: human umbilical vein endothelial cells; NFs: normal human fibroblasts; NKs: normal human keratinocytes; KFs: keloid human fibroblasts; KKs: keloid human keratinocytes; HSFs: hypertrophic-scar-derived fibroblasts; HSKs: hypertrophic scar keratinocytes; Hmyo: myofibroblasts from hypertrophic scar tissue; Wmyo: normal wound myofibroblasts.

## References

[1] Eming S.A., Martin P., Tomic-Canic M. (2014). Wound repair and regeneration: Mechanisms, signaling, and translation. Sci. Transl. Med..

[2] Wilkinson H.N., Hardman M.J. (2020). Wound healing: Cellular mechanisms and pathological outcomes: Cellular Mechanisms of Wound Repair. Open Biol..

[3] Adib Y., Bensussan A., Michel L. (2022). Cutaneous Wound Healing: A Review about Innate Immune Response and Current Therapeutic Applications. Mediat. Inflamm..

[4] Eming S.A., Murray P.J., Pearce E.J. (2021). Metabolic orchestration of the wound healing response. Cell Metab..

[5] Willenborg S., Injarabian L., Eming S.A. (2022). Role of Macrophages in Wound Healing. Cold Spring Harb. Perspect. Biol..

[6] Sgonc R., Gruber J. (2013). Age-related aspects of cutaneous wound healing: A mini-review. Gerontology.

[7] Rodrigues M., Kosaric N., Bonham C.A., Gurtner G.C. (2019). Wound healing: A cellular perspective. Physiol. Rev..

[8] Talbott H.E., Mascharak S., Griffin M., Wan D.C., Longaker M.T. (2022). Wound healing, fibroblast heterogeneity, and fibrosis. Cell Stem Cell.

[9] Tarusha L., Paoletti S., Travan A., Marsich E. (2018). Alginate membranes loaded with hyaluronic acid and silver nanoparticles to foster tissue healing and to control bacterial contamination of non-healing wounds. J. Mater. Sci. Mater. Med..

[10] Raziyeva K., Kim Y., Zharkinbekov Z., Kassymbek K., Jimi S., Saparov A. (2021). Immunology of Acute and Chronic Wound Healing. Biomolecules.

[11] Zhang L., Qin H., Wu Z., Chen W., Zhang G. (2018). Identification of the potential targets for keloid and hypertrophic scar prevention. J. Dermatolog. Treat..

[12] Reinke J.M., Sorg H. (2012). Wound repair and regeneration. Eur. Surg. Res..

[13] Avci P., Sadasivam M., Gupta A., Melo W., Huang Y.-Y., Yin R., Chandran R., Kumar R., Otufowora A., Nyame T. (2013). Animal models of skin disease for drug discovery. Expert Opin. Drug Discov..

[14] Jung E.C., Maibach H.I. (2015). Animal models for percutaneous absorption. J. Appl. Toxicol..

[15] Dellambra E., Odorisio T., D’Arcangelo D., Failla C.M., Facchiano A. (2019). Non-animal models in dermatological research. ALTEX.

[16] Naldaiz-Gastesi N., Bahri O.A., Opez De Munain A.L., Mccullagh K.J.A., Izeta A. (2018). The panniculus carnosus muscle: An evolutionary enigma at the intersection of distinct research fields. J. Anat..

[17] Abdullahi A., Amini-Nik S., Jeschke M.G. (2014). Animal models in burn research. Cell. Mol. Life Sci..

[18] Gottrup F., Ågren M.S., Karlsmark T. (2000). Models for use in wound healing research: A survey focusing on in vitro and in vivo adult soft tissue. Wound Repair Regen..

[19] Dahiya P. (2009). Burns as a model of SIRS. Front. Biosci..

[20] Wong V.W., Sorkin M., Glotzbach J.P., Longaker M.T., Gurtner G.C. (2011). Surgical approaches to create murine models of human wound healing. J. Biomed. Biotechnol..

[21] Lorenz H.P., Longaker M.T. (2008). Wounds: Biology, pathology, and management. Surg. Basic Sci. Clin. Evid..

[22] Pavletic M.M. (2018). Atlas of Small Animal Wound Management and Reconstructive Surgery.

[23] Sullivan T.P., Eaglstein W.H., Davis S.C., Mertz P. (2001). The pig as a model for human wound healing. Wound Repair Regen..

[24] Middelkoop E., Van Den Bogaerdt A.J., Lamme E.N., Hoekstra M.J., Brandsma K., Ulrich M.M.W. (2004). Porcine wound models for skin substitution and burn treatment. Biomaterials.

[25] Summerfield A., Meurens F., Ricklin M.E. (2015). The immunology of the porcine skin and its value as a model for human skin. Mol. Immunol..

[26] Niehues H., Bouwstra J.A., El Ghalbzouri A., Brandner J.M., Zeeuwen P.L.J.M., van den Bogaard E.H. (2018). 3D skin models for 3R research: The potential of 3D reconstructed skin models to study skin barrier function. Exp. Dermatol..

[27] Mathes S.H., Ruffner H., Graf-Hausner U. (2014). The use of skin models in drug development. Adv. Drug Deliv. Rev..

[28] Stamm A., Reimers K., Strauß S., Vogt P., Scheper T., Pepelanova I. (2016). In vitro wound healing assays-state of the art. BioNanoMat.

[29] Groeber F., Holeiter M., Hampel M., Hinderer S., Schenke-Layland K. (2011). Skin tissue engineering-In vivo and in vitro applications. Adv. Drug Deliv. Rev..

[30] Liang C.C., Park A.Y., Guan J.L. (2007). In vitro scratch assay: A convenient and inexpensive method for analysis of cell migration in vitro. Nat. Protoc..

[31] Pinto B.I., Tabor A.J., Stearns D.M., Diller R.B., Kellar R.S. (2016). A Bench-Top In Vitro Wound Assay to Demonstrate the Effects of Platelet-Rich Plasma and Depleted Uranium on Dermal Fibroblast Migration. Appl. Vitr. Toxicol..

[32] Kramer N., Walzl A., Unger C., Rosner M., Krupitza G., Hengstschläger M., Dolznig H. (2013). In vitro cell migration and invasion assays. Mutat. Res.—Rev. Mutat. Res..

[33] Jonkman J.E.N., Cathcart J.A., Xu F., Bartolini M.E., Amon J.E., Stevens K.M., Colarusso P. (2014). An introduction to the wound healing assay using live-cell microscopy. Cell Adhes. Migr..

[34] Riis S., Newman R., Ipek H., Andersen J.I., Kuninger D., Boucher S., Vemuri M.C., Pennisi C.P., Zachar V., Fink T. (2017). Hypoxia enhances the woundhealing potential of adipose-derived stem cells in a novel human primary keratinocyte-based scratch assay. Int. J. Mol. Med..

[35] Yue P.Y.K., Leung E.P.Y., Mak N.K., Wong R.N.S. (2010). A Simplified Method for Quantifying Cell Migration/Wound Healing in 96-Well Plates. J. Biomol. Screen..

[36] Giaever I., Keese C.R. (1991). Micromotion of mammalian cells measured electrically. Proc. Natl. Acad. Sci. USA.

[37] Anwer S., Szászi K. (2020). Measuring Cell Growth and Junction Development in Epithelial Cells Using Electric Cell-Substrate Impedance Sensing (ECIS). Bio-Protocol.

[38] Keese C.R., Wegener J., Walker S.R., Giaever I. (2004). Electrical wound-healing assay for cells in vitro. Proc. Natl. Acad. Sci. USA.

[39] Hundsberger H., Koppensteiner A., Hofmann E., Ripper D., Pflüger M., Stadlmann V., Klein C.T., Kreiseder B., Katzlinger M., Eger A. (2017). A Screening Approach for Identifying Gliadin Neutralizing Antibodies on Epithelial Intestinal Caco-2 Cells. SLAS Discov. Adv. Life Sci. R D.

[40] Pflüger M., Kapuscik A., Lucas R., Koppensteiner A., Katzlinger M., Jokela J., Eger A., Jacobi N., Wiesner C., Hofmann E. (2013). A Combined Impedance and AlphaLISA-Based Approach to Identify Anti-inflammatory and Barrier-Protective Compounds in Human Endothelium. J. Biomol. Screen..

[41] Hung Y.H., Chiu W.C., Fuh S.R., Lai Y.T., Tung T.H., Huang C.C., Lo C.M. (2022). ECIS Based Electric Fence Method for Measurement of Human Keratinocyte Migration on Different Substrates. Biosensors.

[42] Ramasamy S., Bennet D., Kim S. (2014). Drug and bioactive molecule screening based on a bioelectrical impedance cell culture platform. Int. J. Nanomed..

[43] Sun T., Jackson S., Haycock J.W., Macneil S. (2006). Culture of skin cells in 3D rather than 2D improves their ability to survive exposure to cytotoxic agents. J. Biotechnol..

[44] Bhadriraju K., Chen C.S. (2002). Engineering cellular microenvironments to improve cell-based drug testing. Drug Discov. Today.

[45] Antoni D., Burckel H., Josset E., Noel G., Antoni D., Burckel H., Josset E., Noel G. (2015). Three-Dimensional Cell Culture: A Breakthrough in Vivo. Int. J. Mol. Sci..

[46] Pampaloni F., Reynaud E.G., Stelzer E.H.K. (2007). The third dimension bridges the gap between cell culture and live tissue. Nat. Rev. Mol. Cell Biol..

[47] Laplante A.F., Germain L., Auger F.A., Moulin V. (2001). Mechanisms of wound reepithelialization: Hints from a tissue-engineered reconstructed skin to long-standing questions. FASEB J..

[48] El Ghalbzouri A., Hensbergen P., Gibbs S., Kempenaar J., van der Schors R., Ponec M. (2004). Fibroblasts facilitate re-epithelialization in wounded human skin equivalents. Lab. Investig..

[49] Breetveld M., Richters C.D., Rustemeyer T., Scheper R.J., Gibbs S. (2006). Comparison of wound closure after burn and cold injury in human skin equivalents. J. Investig. Dermatol..

[50] Xie Y., Rizzi S.C., Dawson R., Lynam E., Richards S., Leavesley D.I., Upton Z. (2010). Development of a three-dimensional human skin equivalent wound model for investigating novel wound healing therapies. Tissue Eng. Part C. Methods.

[51] Egles C., Garlick J.A., Shamis Y., Turksen K. (2010). Three-Dimensional Human Tissue Models of Wounded Skin. Epidermal Cells: Methods in Molecular Biology.

[52] Blais M., Mottier L., Germain M.-A., Bellenfant S., Cadau S., Berthod F. (2014). Sensory Neurons Accelerate Skin Reepithelialization via Substance P in an Innervated Tissue-Engineered Wound Healing Model. Tissue Eng. Part A.

[53] Langhans S.A. (2018). Three-dimensional in vitro cell culture models in drug discovery and drug repositioning. Front. Pharmacol..

[54] Iyer K., Chen Z., Ganapa T., Wu B.M., Tawil B., Linsley C.S. (2018). Keratinocyte Migration in a Three-Dimensional In Vitro Wound Healing Model Co-Cultured with Fibroblasts. Tissue Eng. Regen. Med..

[55] Graves Id N., Phillips Id C.J., Harding Id K., Graves N. (2021). A narrative review of the epidemiology and economics of chronic wounds. Br. J. Dermatol..

[56] Zhao R., Liang H., Clarke E., Jackson C., Xue M. (2016). Inflammation in chronic wounds. Int. J. Mol. Sci..

[57] Nunan R., Harding K.G., Martin P. (2014). Clinical challenges of chronic wounds: Searching for an optimal animal model to recapitulate their complexity. DMM Dis. Model. Mech..

[58] Holzer-Geissler J.C.J., Schwingenschuh S., Zacharias M., Einsiedler J., Kainz S., Reisenegger P., Holecek C., Hofmann E., Wolff-Winiski B., Fahrngruber H. (2022). Article The Impact of Prolonged Inflammation on Wound Healing. Biomedicines.

[59] Monika P., Chandraprabha M.N., Murthy K.N.C., Rangarajan A., Waiker P.V., Sathish M. (2022). Human primary chronic wound derived fibroblasts demonstrate differential pattern in expression of fibroblast specific markers, cell cycle arrest and reduced proliferation. Exp. Mol. Pathol..

[60] Wall I.B., Moseley R., Baird D.M., Kipling D., Giles P., Laffafian I., Price P.E., Thomas D.W., Stephens P. (2008). Fibroblast dysfunction is a key factor in the non-healing of chronic venous leg ulcers. J. Investig. Dermatol..

[61] Schwarz F., Jennewein M., Bubel M., Holstein J.H., Pohlemann T., Oberringer M. (2013). Soft tissue fibroblasts from well healing and chronic human wounds show different rates of myofibroblasts in vitro. Mol. Biol. Rep..

[62] Caley M., Wall I.B., Peake M., Kipling D., Giles P., Thomas D.W., Stephens P. (2018). Development and characterisation of a human chronic skin wound cell line—Towards an alternative for animal experimentation. Int. J. Mol. Sci..

[63] Maione A.G., Brudno Y., Stojadinovic O., Park L.K., Smith A., Tellechea A., Leal E.C., Kearney C.J., Veves A., Tomic-Canic M. (2015). Three-Dimensional Human Tissue Models That Incorporate Diabetic Foot Ulcer-Derived Fibroblasts Mimic In Vivo Features of Chronic Wounds. Tissue Eng. Part C. Methods.

[64] Ozdogan C.Y., Kenar H., Davun K.E., Yucel D., Doger E., Alagoz S. (2021). An in vitro 3D diabetic human skin model from diabetic primary cells. Biomed. Mater..

[65] Lim I.J., Phan T.T., Song C., Tan W.T.L., Longaker M.T. (2001). Investigation of the influence of keloid-derived keratinocytes on fibroblast growth and proliferation in vitro. Plast. Reconstr. Surg..

[66] Funayama E., Chodon T., Oyama A., Sugihara T. (2003). Keratinocytes Promote Proliferation and Inhibit Apoptosis of the Underlying Fibroblasts: An Important Role in the Pathogenesis of Keloid. J. Investig. Dermatol..

[67] Butler P.D., Ly D.P., Longaker M.T., Yang G.P. (2008). Use of organotypic coculture to study keloid biology. Am. J. Surg..

[68] Suttho D., Mankhetkorn S., Binda D., Pazart L., Humbert P., Rolin G. (2017). 3D modeling of keloid scars in vitro by cell and tissue engineering. Arch. Dermatol. Res..

[69] Limandjaja G.C., van den Broek L.J., Breetveld M., Waaijman T., Monstrey S., de Boer E.M., Scheper R.J., Niessen F.B., Gibbs S. (2018). Characterization of In Vitro Reconstructed Human Normotrophic, Hypertrophic, and Keloid Scar Models. Tissue Eng. Part C Methods.

[70] Limandjaja G.C., van den Broek L.J., Waaijman T., Breetveld M., Monstrey S., Scheper R.J., Niessen F.B., Gibbs S. (2018). Reconstructed human keloid models show heterogeneity within keloid scars. Arch. Dermatol. Res..

[71] Monsuur H.N., Boink M.A., Weijers E.M., Roffel S., Breetveld M., Gefen A., van den Broek L.J., Gibbs S. (2016). Methods to study differences in cell mobility during skin wound healing in vitro. J. Biomech..

[72] Limandjaja G.C., Waaijman T., Roffel S., Niessen F.B., Gibbs S. (2019). Monocytes co-cultured with reconstructed keloid and normal skin models skew towards M2 macrophage phenotype. Arch. Dermatol. Res..

[73] Phan T.-T., Sun L., Bay B.-H., Chan S.-Y., Lee S.-T. (2003). Dietary Compounds Inhibit Proliferation and Contraction of Keloid and Hypertrophic Scar-Derived Fibroblasts In Vitro. J. Trauma Inj. Infect. Crit. Care.

[74] Wang J., Dodd C., Shankowsky H.A., Scott P.G., Tredget E.E. (2008). Deep dermal fibroblasts contribute to hypertrophic scarring. Lab. Investig..

[75] De Felice B., Ciarmiello L.F., Mondola P., Damiano S., Seru R., Argenziano C., Nacca M., Santoriello M., Garbi C. (2007). Differential p63 and p53 Expression in Human Keloid Fibroblasts and Hypertrophic Scar Fibroblasts. DNA Cell Biol..

[76] De Felice B., Garbi C., Santoriello M., Santillo A., Wilson R.R. (2009). Differential apoptosis markers in human keloids and hypertrophic scars fibroblasts. Mol. Cell. Biochem..

[77] Zhang G.Y., Cheng T., Zheng M.H., Yi C.G., Pan H., Li Z.J., Chen X.L., Yu Q., Jiang L.F., Zhou F.Y. (2009). Activation of peroxisome proliferator-activated receptor-inhibits transforming growth factor-1 induction of connective tissue growth factor and extracellular matrix in hypertrophic scar Wbroblasts in vitro. Arch Dermatol Res.

[78] Lee J.S., Kim J.S., Lee J.W., Choi K.Y., Yang J.D., Cho B.C., Oh E.J., Kim T.J., Ko U.H., Shin J.H. (2019). Effect of Keratinocytes on Myofibroblasts in Hypertrophic Scars. Aesthetic Plast. Surg..

[79] Moulin V., Larochelle S., Langlois C., Thibault I., Lopez-Vallé C.A., Roy M. (2004). Normal skin wound and hypertrophic scar myofibroblasts have differential responses to apoptotic inductors. J. Cell. Physiol..

[80] Linge C., Richardson J., Vigor C., Clayton E., Hardas B., Rolfe K.J. (2005). Hypertrophic scar cells fail to undergo a form of apoptosis specific to contractile collagen—The role of tissue transglutaminase. J. Investig. Dermatol..

[81] Younai S., Nichter L.S., Wellisz T., Reinisch J., Nimni M.E., Tuan T.L., Tredget E.E. (1994). Modulation of collagen synthesis by transforming growth factor-β in keloid and hypertrophic scar fibroblasts. Ann. Plast. Surg..

[82] Simon F., Bergeron D., Larochelle S., Lopez-Vallé C.A., Genest H., Armour A., Moulin V.J. (2012). Enhanced secretion of TIMP-1 by human hypertrophic scar keratinocytes could contribute to fibrosis. Burns.

[83] Bellemare J., Roberge C.J., Bergeron D., Lopez-Vallé C.A., Roy M., Moulin V.J. (2005). Epidermis promotes dermal fibrosis: Role in the pathogenesis of hypertrophic scars. J. Pathol..

[84] Lafosse A., Dufeys C., Beauloye C., Horman S., Dufrane D. (2016). Impact of hyperglycemia and low oxygen tension on adipose-derived stem cells compared with dermal fibroblasts and keratinocytes: Importance for wound healing in type 2 diabetes. PLoS ONE.

[85] Lan C.C.E., Liu I.H., Fang A.H., Wen C.H., Wu C.S. (2008). Hyperglycaemic conditions decrease cultured keratinocyte mobility: Implications for impaired wound healing in patients with diabetes. Br. J. Dermatol..

[86] Pan F., Guo R., Cheng W., Chai L., Wang W., Cao C., Li S. (2015). High glucose inhibits ClC-2 chloride channels and attenuates cell migration of rat keratinocytes. Drug Des. Devel. Ther..

[87] Wright C.S., Berends R.F., Flint D.J., Martin P.E.M. (2013). Cell motility in models of wounded human skin is improved by Gap27 despite raised glucose, insulin and IGFBP-5. Exp. Cell Res..

[88] Ueck C., Volksdorf T., Houdek P., Vidal-Y-Sy S., Sehner S., Ellinger B., Lobmann R., Larena-Avellaneda A., Reinshagen K., Ridderbusch I. (2017). Comparison of in-vitro and ex-vivo wound healing assays for the investigation of diabetic wound healing and demonstration of a beneficial effect of a triterpene extract. PLoS ONE.

[89] Finnson K.W., McLean S., Di Guglielmo G.M., Philip A. (2013). Dynamics of Transforming Growth Factor Beta Signaling in Wound Healing and Scarring. Adv. Wound Care.

[90] Mokos Z.B., Jović A., Grgurević L., Dumić-Čule I., Kostović K., Čeović R., Marinović B. (2017). Current Therapeutic Approach to Hypertrophic Scars. Front. Med..

[91] Robles D.T., Berg D. (2007). Abnormal wound healing: Keloids. Clin. Dermatol..

[92] Limandjaja G.C., Niessen F.B., Scheper R.J., Gibbs S. (2020). The Keloid Disorder: Heterogeneity, Histopathology, Mechanisms and Models. Front. Cell Dev. Biol..

[93] Supp D.M. (2019). Animal Models for Studies of Keloid Scarring. Adv. Wound Care.

[94] Sunaga A., Kamochi H., Sarukawa S., Uda H., Sugawara Y., Asahi R., Chi D., Nakagawa S., Kanayama K., Yoshimura K. (2017). Reconstitution of Human Keloids in Mouse Skin. Plast. Reconstr. Surg. Glob. Open.

[95] Polo M., Kim Y.J., Kucukcelebi A., Hayward P.G., Ko F., Robson M.C. (1998). An in vivo model of human proliferative scar. J. Surg. Res..

[96] Shetlar M.R., Shetlar C.L., Kischer C.W., Pindur J. (1991). Implants of keloid and hypertrophic scars into the athymic nude mouse: Changes in the glycosaminoglycans of the implants. Connect. Tissue Res..

[97] Wang H., Luo S. (2013). Establishment of an animal model for human keloid scars using tissue engineering method. J. Burn Care Res..

[98] Diegelmann R.F., Cohen I.K., McCoy B.J. (1979). Growth kinetics and collagen synthesis of normal skin, normal scar and keloid fibroblasts in vitro. J. Cell. Physiol..

[99] Cohen I.K., Diegelmann R.F., McCoy B. (1977). Elevated collagen synthesis in cultured keloid fibroblasts. Surg. Forum.

[100] Kischer C.W., Wagner H.N., Pindur J., Holubec H., Jones M., Ulreich J.B., Scuderi P. (1989). Increased fibronectin production by cell lines from hypertrophic scar and keloid. Connect. Tissue Res..

[101] Babu M., Diegelmann R., Oliver N. (1989). Fibronectin is overproduced by keloid fibroblasts during abnormal wound healing. Mol. Cell. Biol..

[102] Oliver N., Babu M., Diegelmann R. (1992). Fibronectin gene transcription is enhanced in abnormal wound healing. J. Investig. Dermatol..

[103] Meyer L.J.M., Egbert B.M., Shuster S., Stern R., Russell S.B., Russell J.D., Trupin J.S. (2003). Reduced Hyaluronan in Keloid Tissue and Cultured Keloid Fibroblasts. J. Investig. Dermatol..

[104] Alaish S.M., Yager D.R., Diegelmann R.F., Cohen I.K. (1995). Hyaluronic acid metabolism in keloid fibroblasts. J. Pediatr. Surg..

[105] Clore J.N., Cohen I.K., Diegelmann R.F. (1979). Quantitative assay of types I and III collagen synthesized by keloid biopsies and fibroblasts. Biochim. Biophys. Acta—Gen. Subj..

[106] Russell J.D., Witt W.S. (1976). Cell size and growth characteristics of cultured fibroblasts isolated from normal and keloid tissue. Plast. Reconstr. Surg..

[107] Oku T., Takigawa M., Yamada M. (1987). Cell proliferation kinetics of cultured human keratinocytes and fibroblasts measured using a monoclonal antibody. Br. J. Dermatol..

[108] MANCINI R.E., QUAIFE J.V. (1962). Histogenesis of experimentally produced keloids. J. Investig. Dermatol..

[109] Matsuoka L.Y., Uitto J., Wortsman J., Abergel R.P., Dietrich J. (1988). Ultrastructural Characteristics of Keloid Fibroblasts. Am. J. Dermatopathol..

[110] Calderon M., Lawrence W.T., Banes A.J. (1996). Increased proliferation in keloid fibroblasts wounded in vitro. J. Surg. Res..

[111] Maas-Szabowski N., Stark H.-J., Fusenig N.E. (2000). Keratinocyte Growth Regulation in Defined Organotypic Cultures Through IL-1-Induced Keratinocyte Growth Factor Expression in Resting Fibroblasts. J. Investig. Dermatol..

[112] Maas-Szabowski N., Shimotoyodome A., Fusenig N.E. (1999). Keratinocyte growth regulation in fibroblast cocultures via a double paracrine mechanism. J. Cell Sci..

[113] Szabowski A., Maas-Szabowski N., Andrecht S., Kolbus A., Schorpp-Kistner M., Fusenig N.E., Angel P. (2000). c-Jun and JunB antagonistically control cytokine-regulated mesenchymal-epidermal interaction in skin. Cell.

[114] Garner W.L. (1998). Epidermal Regulation of Dermal Fibroblast Activity. Plast. Reconstr. Surg..

[115] Lim I.J., Phan T.T., Bay B.H., Qi R., Huynh H.-T., Tan W.T.L., Lee S.T., Longaker M.T. (2002). Fibroblasts cocultured with keloid keratinocytes: Normal fibroblasts secrete collagen in a keloidlike manner. Am. J. Physiol.—Cell Physiol..

[116] Phan T.-T., Lim I.J., Bay B.-H., Qi R., Huynh H.T., Lee S.-T., Longaker M.T. (2002). Differences in collagen production between normal and keloid-derived fibroblasts in serum-media co-culture with keloid-derived keratinocytes. J. Dermatol. Sci..

[117] Hahn J.M., Glaser K., McFarland K.L., Aronow B.J., Boyce S.T., Supp D.M. (2013). Keloid-derived keratinocytes exhibit an abnormal gene expression profile consistent with a distinct causal role in keloid pathology. Wound Repair Regen..

[118] Rosen D.J., Patel M.K., Freeman K., Weiss P.R. (2007). A primary protocol for the management of ear keloids: Results of excision combined with intraoperative and postoperative steroid injections. Plast. Reconstr. Surg..

[119] Golladay E.S. (1988). Treatment of keloids by single intraoperative perilesional injection of repository steroid. South. Med. J..

[120] Hietanen K.E., Järvinen T.A., Huhtala H., Tolonen T.T., Kuokkanen H.O., Kaartinen I.S. (2019). Treatment of keloid scars with intralesional triamcinolone and 5-fluorouracil injections—A randomized controlled trial. J. Plast. Reconstr. Aesthetic Surg..

[121] Darougheh A., Asilian A., Shariati F. (2009). Intralesional triamcinolone alone or in combination with 5-fluorouracil for the treatment of keloid and hypertrophic scars. Clin. Exp. Dermatol..

[122] Apikian M., Goodman G. (2004). Intralesional 5-fluorouracil in the treatment of keloid scars. Australas. J. Dermatol..

[123] Khalid F.A., Mehrose M.Y., Saleem M., Yousaf M.A., Mujahid A.M., Rehman S.U., Ahmad S., Tarar M.N. (2019). Comparison of efficacy and safety of intralesional triamcinolone and combination of triamcinolone with 5-fluorouracil in the treatment of keloids and hypertrophic scars: Randomised control trial. Burns.

[124] Syed F., Bayat A. (2013). Superior effect of combination vs. single steroid therapy in keloid disease: A comparative in vitro analysis of glucocorticoids. Wound Repair Regen..

[125] Huang L., Cai Y.J., Lung I., Leung B.C.S., Burd A. (2013). A study of the combination of triamcinolone and 5-fluorouracil in modulating keloid fibroblasts in vitro. J. Plast. Reconstr. Aesthetic Surg..

[126] Gupta S., Kalra A. (2002). Efficacy and safety of intralesional 5-fluorouracil in the treatment of keloids. Dermatology.

[127] Fitzpatrick R.E. (1999). Treatment of inflamed hypertrophic scars using intralesional 5-FU. Dermatol. Surg..

[128] Son Y., Phillips E.O.N., Price K.M., Rosenberg L.Z., Stefanovic B., Wolfe C.M., Shaath T.S., Om A., Cohen G.F., Gunjan A. (2020). Treatment of keloids with a single dose of low-energy superficial X-ray radiation to prevent recurrence after surgical excision: An in vitro and in vivo study. J. Am. Acad. Dermatol..

[129] Rössler S., Nischwitz S.P., Luze H., Holzer-Geissler J.C.J., Zrim R., Kamolz L.P. (2022). In Vivo Models for Hypertrophic Scars&mdash;A Systematic Review. Medicina.

[130] van den Broek L.J., Limandjaja G.C., Niessen F.B., Gibbs S. (2014). Human hypertrophic and keloid scar models: Principles, limitations and future challenges from a tissue engineering perspective. Exp. Dermatol..

[131] Ramos M.L.C., Gragnani A., Ferreira L.M. (2008). Is there an ideal animal model to study hypertrophic scarring?. J. Burn Care Res..

[132] Zhu K.Q., Engrav L.H., Tamura R.N., Cole J.A., Muangman P., Carrougher G.J., Gibran N.S. (2004). Further similarities between cutaneous scarring in the female, red Duroc pig and human hypertrophic scarring. Burns.

[133] Blackstone B.N., Kim J.Y., McFarland K.L., Sen C.K., Supp D.M., Bailey J.K., Powell H.M. (2017). Scar formation following excisional and burn injuries in a red Duroc pig model. Wound Repair Regen..

[134] Gallant-Behm C.L., Hart D.A. (2006). Genetic analysis of skin wound healing and scarring in a porcine model. Wound Repair Regen..

[135] Nischwitz S.P., Fink J., Schellnegger M., Luze H., Bubalo V., Tetyczka C., Roblegg E., Holecek C., Zacharias M., Kamolz L. (2023). The Role of Local Inflammation and Hypoxia in the Formation of Hypertrophic Scars—A New Model in the Duroc Pig. Int. J. Mol. Sci..

[136] Honardoust D., Kwan P., Momtazi M., Ding J., Tredget E.E. (2013). Novel methods for the investigation of human hypertrophic scarring and other dermal fibrosis. Methods Mol. Biol..

[137] Varkey M., Ding J., Tredget E.E. (2011). Differential collagen-glycosaminoglycan matrix remodeling by superficial and deep dermal fibroblasts: Potential therapeutic targets for hypertrophic scar. Biomaterials.

[138] Derderian C.A., Bastidas N., Lerman O.Z., Bhatt K.A., Lin S.E., Voss J., Holmes J.W., Levine J.P., Gurtner G.C. (2005). Mechanical strain alters gene expression in an in vitro model of hypertrophic scarring. Ann. Plast. Surg..

[139] Van Den Broek L.J., Niessen F.B., Scheper R.J., Gibbs S. (2012). Development, validation, and testing of a human tissue engineered hypertrophic scar model. ALTEX.

[140] Li J., Wang J., Wang Z., Xia Y., Zhou M., Zhong A., Sun J. (2020). Experimental models for cutaneous hypertrophic scar research. Wound Repair Regen..

[141] Ogawa R. (2017). Keloid and hypertrophic scars are the result of chronic inflammation in the reticular dermis. Int. J. Mol. Sci..

[142] Trottier V., Marceau-Fortier G., Germain L., Vincent C., Fradette J. (2008). IFATS Collection: Using Human Adipose-Derived Stem/Stromal Cells for the Production of New Skin Substitutes. Stem Cells.

[143] Bellas E., Seiberg M., Garlick J., Kaplan D.L. (2012). In vitro 3D Full-Thickness Skin-Equivalent Tissue Model Using Silk and Collagen Biomaterials. Macromol. Biosci..

[144] Monfort A., Soriano-Navarro M., García-Verdugo J.M., Izeta A. (2013). Production of human tissue-engineered skin trilayer on a plasma-based hypodermis. J. Tissue Eng. Regen. Med..

[145] Gimble J.M., Katz A.J., Bunnell B.A. (2007). Adipose-derived stem cells for regenerative medicine. Circ. Res..

[146] Satija N.K., Gurudutta G.U., Sharma S., Afrin F., Gupta P., Verma Y.K., Singh V.K., Tripathi R.P. (2007). Mesenchymal stem cells: Molecular targets for tissue engineering. Stem Cells Dev..

[147] Huber B., Link A., Linke K., Gehrke S.A., Winnefeld M., Kluger P.J. (2016). Integration of Mature Adipocytes to Build-Up a Functional Three-Layered Full-Skin Equivalent. Tissue Eng.—Part C Methods.

[148] Huber B., Borchers K., Tovar G.E.M., Kluger P.J. (2015). Methacrylated gelatin and mature adipocytes are promising components for adipose tissue engineering. J. Biomater. Appl..

[149] Ataç B., Wagner I., Horland R., Lauster R., Marx U., Tonevitsky A.G., Azar R.P., Lindner G. (2013). Skin and hair on-a-chip: In vitro skin models versus ex vivo tissue maintenance with dynamic perfusion. Lab Chip.

[150] Vidal S.E.L., Tamamoto K.A., Nguyen H., Abbott R.D., Cairns D.M., Kaplan D.L. (2019). 3D biomaterial matrix to support long term, full thickness, immuno-competent human skin equivalents with nervous system components. Biomaterials.

[151] Groeber F., Engelhardt L., Lange J., Kurdyn S., Schmid F.F., Rücker C., Mielke S., Walles H., Hansmann J. (2016). A first vascularized skin equivalent for as an alternative to animal experimentation. ALTEX.

[152] Kim B.S., Gao G., Kim J.Y., Cho D. (2019). 3D Cell Printing of Perfusable Vascularized Human Skin Equivalent Composed of Epidermis, Dermis, and Hypodermis for Better Structural Recapitulation of Native Skin. Adv. Healthc. Mater..

[153] Matai I., Kaur G., Seyedsalehi A., McClinton A., Laurencin C.T. (2020). Progress in 3D bioprinting technology for tissue/organ regenerative engineering. Biomaterials.

[154] Murphy S.V., Atala A. (2014). 3D bioprinting of tissues and organs. Nat. Biotechnol..

[155] Abaci H.E., Guo Z., Coffman A., Gillette B., Lee W.H., Sia S.K., Christiano A.M. (2016). Human Skin Constructs with Spatially Controlled Vasculature Using Primary and iPSC-Derived Endothelial Cells. Adv. Healthc. Mater..

[156] Pupovac A., Senturk B., Griffoni C., Maniura-Weber K., Rottmar M., McArthur S.L. (2018). Toward Immunocompetent 3D Skin Models. Adv. Healthc. Mater..

[157] Ouwehand K., Spiekstra S.W., Waaijman T., Scheper R.J., de Gruijl T.D., Gibbs S. (2011). Technical Advance: Langerhans cells derived from a human cell line in a full-thickness skin equivalent undergo allergen-induced maturation and migration. J. Leukoc. Biol..

[158] Kühbacher A., Henkel H., Stevens P., Grumaz C., Finkelmeier D., Burger-Kentischer A., Sohn K., Rupp S. (2017). Central Role for Dermal Fibroblasts in Skin Model Protection against Candida albicans. J. Infect. Dis..

[159] Chau D.Y.S., Johnson C., Macneil S., Haycock J.W., Ghaemmaghami A.M. (2013). The development of a 3D immunocompetent model of human skin. Biofabrication.

[160] Bechetoille N., Dezutter-Dambuyant C., Damour O., André V., Orly I., Perrier E. (2007). Effects of Solar Ultraviolet Radiation on Engineered Human Skin Equivalent Containing Both Langerhans Cells and Dermal Dendritic Cells. Tissue Eng..

[161] Schellenberger M.T., Bock U., Hennen J., Groeber-Becker F., Walles H., Blömeke B. (2019). A coculture system composed of THP-1 cells and 3D reconstructed human epidermis to assess activation of dendritic cells by sensitizing chemicals after topical exposure. Toxicol. Vitr..

[162] Takahashi K., Tanabe K., Ohnuki M., Narita M., Ichisaka T., Tomoda K., Yamanaka S. (2007). Induction of pluripotent stem cells from adult human fibroblasts by defined factors. Cell.

[163] Loh Y.H., Agarwal S., Park I.H., Urbach A., Huo H., Heffner G.C., Kim K., Miller J.D., Ng K., Daley G.Q. (2009). Generation of induced pluripotent stem cells from human blood. Blood.

[164] Guo Z., Higgins C.A., Gillette B.M., Itoh M., Umegaki N., Gledhill K., Sia S.K., Christiano A.M. (2013). Building a microphysiological skin model from induced pluripotent stem cells. Stem Cell Res. Ther..

[165] Kashpur O., Smith A., Gerami-Naini B., Maione A.G., Calabrese R., Tellechea A., Theocharidis G., Liang L., Pastar I., Tomic-Canic M. (2019). Differentiation of diabetic foot ulcer–derived induced pluripotent stem cells reveals distinct cellular and tissue phenotypes. FASEB J..

[166] Ng W.L., Wang S., Yeong W.Y., Naing M.W. (2016). Skin Bioprinting: Impending Reality or Fantasy?. Trends Biotechnol..

[167] Dos Santos M., Metral E., Boher A., Rousselle P., Thepot A., Damour O. (2015). In vitro 3-D model based on extending time of culture for studying chronological epidermis aging. Matrix Biol..

